# Reviews of Books

**Published:** 1922-08

**Authors:** 


### REVIEWS OF BOOKS.

**SEX PROBLEMS IN WOMEN.** By A. C. Magian, M.D. Pp. 219. (William Heinemann (Medical Books), Ltd.; 12s. 6d. net.)

This book is intended as a guide to the general practitioner in dealing with the important and complicated subjects of which it treats. A practitioner who studies it will be able to give advice to his patients on very orthodox lines. Whether this advice will be as helpful as is possible is another matter. The book being intended for medical readers, it is not quite clear why the author has thought it needful to include a chapter on the anatomy and physiology of the female generative apparatus. But the author lays much stress upon the paramount necessity of making a full and careful physical examination as a preliminary in every case. This is well, for there has always been a tendency to overlook, or to shirk, this most necessary examination, and this tendency is, perhaps, accentuated at the present day.

But anatomy and physiology do not contain the whole story. Psychology is of even greater importance. No one is competent to elucidate these problems without a full knowledge of the recent advances made in this line of practice by the theories of the unconscious mind. It is not necessary that every practitioner should be prepared to carry out treatment in this direction. But he cannot do his best for his patients unless he understands enough of the psychology of the matter to know when to send them to a specialist in psychical treatment. Whether we adopt the theories of Freud, or of any other leader, it is established that, in this connection, repressed mental conflict is of supreme importance. But the author gives hardly any information on this head. Indeed, it would almost seem as if he had not broken down his own sexual repressions. The importance of the sex instinct is somewhat underrated. In treating of masturbation, it is not clearly pointed out that any harm which may result is due not so much to the act itself as to the patient's idea that it is physically harmful or morally wrong.

What may be termed the "social" aspects of the book are of much interest. On the question of birth control the author takes a very sensible line. He discusses the various methods. But he does not condemn the practice of *coitus interruptus* with sufficient severity; of all the ordinary methods, it is the one most deleterious to both partners. He has excellent remarks upon the question of prostitution. He says that certain classes of persons—*e.g.*, consumptives, inebriates, habitual criminals, &c.—should not be allowed to marry. Many people will agree. But he goes on to say that they should not be allowed to beget children, without suggesting any practicable way in which this desirable end can be attained.

Severe remarks are made upon the effect of the modern "feminist" movement. These will not meet with general approval. The author seems inclined to take too Victorian a view, and the woman's side of the question is hardly recognised enough. The perfect book on this difficult subject has yet to be written. It will, perhaps, have to be produced by a man and a woman conjointly.

The chapters on puberty and on the menopause are excellent. The whole book is welcome, as an honest attempt to face problems boldly. It will be of special service if it induces readers to consult the great works of Kraft-Ebing, Havelock Ellis and Bloch, and if it leads them to make first-hand acquaintance with the researches of the leaders in the "new psychology."

---

**ARTIFICIAL LIMBS AND AMPUTATION STUMPS: A PRACTICAL HANDBOOK.** By E. Muirhead Little, F.R.C.S. (Eng.), Consulting Surgeon to the Royal National Orthopædic Hospital, &c., London. (H. K. Lewis & Co.; 267 illustrations, pp. 319, viii; 18s. net.)

The war has taught us much in regard to amputation stumps and artificial limbs, as it has done in many other branches of surgery. The present volume is from the pen of an orthopædic surgeon who has had exceptional experience It contains his conclusions after six years' work in the treatment of amputation stumps at the Royal National Orthopædic Hospital, various Red Cross Hospitals and in the prescription and supervision of prothesis at the Queen Mary Convalescent Auxiliary Hospital, Roehampton. According to his statistics, 41,300 British soldiers (exclusive of overseas forces) lost limbs in the war, and about 25,000 of these passed through Roehampton, thus giving him a unique experience. This volume consists of 21 chapters and an appendix containing specifications for artificial limbs and directions for making and reports on certalmid sockets. Each chapter is complete in itself and all full of interest and information, but one or two stand out, especially Chapter X, on amputations of both hands and mutilations of the hand, which shows in great detail what can be done for those who have lost anything from a few fingers up to both arms at the shoulder. In Chapter XIII a comparison is made by means of cinematograph pictures between the normal gait and the gait with an artificial leg. Chapter XXI. is on the re-education of the amputated and is the paper in which the author introduced the discussion on this subject at the Medical Society of London. The book is profusely illustrated throughout. The bulk of the illustrations are from original photographs and a few are from instrument makers' catalogues. The mechanism of the various protheses is shown in great detail. The aluminium limb seems to be the one in favour at the moment, but it has still to stand the test of time. This handbook gives a complete description of the British methods of dealing with amputation stumps and will doubtless become the standard text-book on the subject, and should be in the library of all those who practise the art of surgery.—T. P. N.

---

**THE VENEREAL CLINIC.** By several writers. Edited by Ernest R. T. Clarkson, M.A., Cantab, M.R.C.S., L.R.C.P. (John Bale, Sons & Danielsson, Ltd.; 25s. net.)

This book aims at fulfilling a double purpose; it is designed to appeal to two groups of readers—the medical man and the layman. It is open to question whether the aim of the editor is best met in a single volume such as this. There is material here for two books, and it would perhaps have been better to separate the two parts. The student who seeks a knowledge of the medical aspects of venereal disease has neither time nor inclination to interest himself in the social problems which they involve, and the layman who is interested in the sociological aspect of venereal disease is hardly likely to appreciate the chapters on the medical aspect which are here provided. Both portions of the book are well written, and the views put forward are those of men and women who have earned the right to speak in the school of experience. It is obvious that the importance of gonorrhœa bulks more largely in the editor's view than that of syphilis; it is unfortunate that the latter subject has been dealt with in a rather condensed manner, for what is said is so valuable that the reader's appetite is whetted for more. As a guide to the diagnosis and treatment of all manifestations of syphilis and gonorrhœa upon modern lines, the book can be recommended to the student and practitioner. The black-and-white illustrations are excellent and the coloured plates are instructive, though they cannot be described in their tinting as absolutely true to life. Great stress is rightly laid upon the importance of tests of cure; the lines laid down are strongly emphasised, and if they are followed should help to prevent much misfortune and disaster in the future.

The chapter upon the equipment and arrangement of a venereal clinic is idealistic rather than practical. Admirable as are the suggested arrangements, they are unlikely to find unqualified support among boards of management, whose zeal has always to be tempered with financial discretion. In the sociological section an impartial and judicial account is given of the tenets of the two prominent anti-venereal societies, whose constant bickering is in danger of bringing the whole subject of venereal control into disrepute. The chapters upon the relation of the medical man to his patients and upon legislation and the practitioner are stimulating and valuable, and the advice given upon the position of the doctor in the witness-box is clear and helpful. The editor is to be congratulated on a sound book, but his appeal would probably meet with a greater response had he not attempted to kill two birds with one stone.—C. E. S.

---

**THE WHITE CROSS OF ST. JOHN.** By Colonel Robert J. Blackham, C.B., C.M.G., M.D. Fourth edition. (Dale, Reynolds & Co.; 3s. 6d. net.)

**TROPICAL HOME NURSING.** By Colonel Robert J. Blackham, C.B., C.M.G., M.D. Thirteenth edition. (Dale, Reynolds & Co.; 2s. net.)

Two little books by the indefatigable Colonel Blackham are before us. "The White Cross of St. John" is an admirable collation, descriptive of the history of the Order and of the work of the St. John Ambulance Association, culled from various sources, and much of it personal recitation of the author's own experiences. Enthusiasts will find it a very valuable medium of propaganda, and the ingenuous and sympathetic personal note will find a wide circle of admirers. The "Notable Ambulance Dates and Facts" included in the booklet, and compiled by Mr. N. Corbet-Fletcher, give added interest.

The other booklet is "Tropical Home Nursing," which appears as a thirteenth edition. The author advises students preparing for examinations to use this book in conjunction with his "Catechism of Home Nursing," and hopes "that this thirteenth edition may continue to merit the cordial reception which has been given to its predecessors, not only by St. John and Red Cross pupils, but by untrained persons called upon to nurse cases of illness at outposts of the Empire." We hope so, too. It is a very comprehensive and ambitious little book, and admirably suited for the courses of instruction for which it is designed.—A. M.

---

**LIVERPOOL SOCIAL WORKERS' HANDBOOK.** (Gledsdale and Jennings, Ltd., North John Street, Liverpool; 2s. 6d. net.)

This handbook, issued by the Liverpool Council of Voluntary Aid, has now run into a fourth edition. Once again it deserves every welcome, for its two hundred odd pages are packed with an amount of information which will probably surprise every inhabitant of the city not already acquainted with the number and inter-relation of the social agencies in its midst. One is tempted to feel that more of the real life of a great modern city is contained in this book than in at least half the columns of the local newspaper. The extent to which co-ordination between voluntary and public bodies has grown may be judged by the statement that "nearly a third of the Liverpool voluntary agencies are now in receipt of public money." The different institutions and agencies are clearly grouped. To avoid overlapping, which the Council was formed to prevent, information concerning the various Acts affecting social administration has been omitted, since this can be found in "Public Services" (price 2s.), issued by the National Council of Social Service. The price of the Liverpool Council's handbook is modest and the book is its own advertisement.—O.B.

